# Editorial: Community series in ethics in psychiatry and psychotherapy — Volume II

**DOI:** 10.3389/fpsyt.2023.1199083

**Published:** 2023-05-03

**Authors:** Cynthia M. A. Geppert, Rebecca Weintraub Brendel, Manuel Trachsel

**Affiliations:** ^1^School of Medicine, University of New Mexico, Albuquerque, NM, United States; ^2^Alden March Bioethics Institute, Albany Medical College, Albany, NY, United States; ^3^Center for Bioethics, Harvard Medical School, Boston, MA, United States; ^4^Harvard Medical School, Massachusetts General Hospital, Boston, MA, United States; ^5^Medical Faculty, University of Basel, Basel, Switzerland; ^6^Clinical Ethics Unit, University Hospital of Basel, Basel, Switzerland; ^7^Clinical Ethics Unit, University Psychiatric Clinic Basel, Basel, Switzerland

**Keywords:** ethics, psychiatry, psychotherapy, mental health, autonomy

A multidisciplinary group of scholars provided significant contributions to Volume I of the *Community Series in Ethics in Psychiatry and Psychotherapy* (https://www.frontiersin.org/research-topics/11564/ethics-in-psychiatry-and-psychotherapy).

The positive response to that collection resulted in a call for a second volume. Volume II continues the substantive ethical scholarship of its predecessor. The authors of these four articles represent a variety of methods, national representation, and topics that display the diversity of academic work in Ethics in Psychiatry and Psychotherapy. Two of the articles cover the classic topics of decisional capacity and assertive care from new perspectives and two feature discussions of the contemporary issues related to palliative care psychiatry and abortion laws.

Decision-making capacity evaluations are among the most common requests consultation-liaison psychiatrists receive from inpatient medical and surgical teams (1). The empirical ethics article from Kane et al. fills a gap in the qualitative literature with their report of semi-structured interviews with consultation-liaison psychiatrists working in general medical hospitals in England, Scotland, and New Zealand. Participants were asked to describe their experience of complex decision-making capacity assessments that presented both ethical and clinical challenges. A unique feature of the article is the narrative from consulting psychiatrists of how they managed these challenges. Thematic analysis of the interviews found four main categories of difficulty: discerning whether the patient's decision reflects their authentic values or dimensions of psychiatric or medical illness; appropriately applying ethical principles to the assessment outcomes; eliding personal bias in assessment of decision-making capacity; and problems in the process of assessment. Contrary to presumptions that psychiatrists would manifest conceits of paternalism and certitude, the respondents expressed epistemic humility and awareness of their own limits. The overarching lesson learned from the study is salutary for educators and mental health practitioners alike: in unraveling difficult decision-making capacity cases, ethics and psychiatry are entrained and expertise in both strands is necessary to principled resolution.

In the contribution on assertive care by Liégeois, the issue of decision-making capacity is again a central factor. The composite case study illustrates the relational grounding of the conceptual analysis. The author formulates the foundational ethical tension in mental health as that between the professionals' duty to respect the autonomy of patients with mental illness and to simultaneously safeguard them from harm. He draws on his service on the ethics committee of a large mental health care organization in Belgium to operationalize his thinking. Liégeois proposes a dimensional framework in which the two constituents are the care-users measure of decision-making capacity and the strength and certitude of the care-professionals concern for serious and imminent harm. The juxtaposition of these two variables creates six levels of ethically justifiable assertive care when the care-user possess some degree of decision-making capacity and four other levels when they are incapacitated. While striving to bring a more objective lens to what are often viewed as subjective decisions, each level remains relationally contextualized to prioritize the dignity and self-determination of the care-user and to preserve the trustworthiness of the treatment alliance whenever possible. Not unlike the sliding scale of capacity determination (2), more assertive interventions are justified when care-users without decision-making capacity pose a high risk of danger to self or others.

The final two articles in this volume explore current issues in mental health ethics. Moureau et al. apply the method of scoping review (3) to survey literature on the topic of end-of-life care for persons with serious and persistent mental illness (SPMI). Palliative care in psychiatry arose in recognition that many individuals with SPMI such as schizophrenia and anorexia nervosa suffer multiple medical comorbidities and a reduced life-expectancy in addition to the often heavy burden of their chronic and severe mental illness symptoms, cognitive impairment, and psychosocial dysfunction (4). The authors point out that although there is a growing literature on palliative care psychiatry for this cohort, there has been far less attention to the specific ethical aspects of providing that care. This is the purpose of their scoping review analyzing ethical questions and issues related to the care of persons with SPMI and life-limiting illness using fundamental ethical principles, virtues, and values in health care such as compassion and respect for dignity. Concerns related to decision-making capacity and how it impacts patient autonomy; social justice evidenced in the lower quality of medical care persons with SPMI historically receive are critiqued. A particularly salient aspect of the review is the focus on the dual and overlapping stigma individuals with SPMI frequently experience at the end of life and the need to incorporate the perspective of these persons into research to challenge this bias. Finally, literature on the controversy surrounding the application of futility determinations and the ethical justification for medically assisted dying in individuals with SPMI is studied in a sensitive and balanced manner.

Tobón et al. are a group of American scholars who examine the impact on women's mental and physical health of the 2022 U. S. Supreme Court decision in *Dobbs v. Jackson* that reversed the access to abortion granted under *Roe v. Wade* and the cascade of restrictive state laws passed in its wake.

The article highlights the significance of the Supreme Court's ruling and state legislation in several ways. The authors provide a data-informed appraisal of its clinical, educational, societal, research, and policy implications. They underscore that these often draconian statues will have the most adverse effect on already disadvantaged populations widening socio-economic inequities and deepening health care disparities such as maternal and infant mortality. Tobón et al. conclude that beyond “the physical morbidity, the psychological sequelae of carrying a forced pregnancy to term will lead to an even greater burden of maternal mental illness, exacerbating the already existing maternal mental health crisis”.

Together with the articles in Volume I, this collection of thoughtful contributions on *Ethics in Psychiatry and Psychotherapy* demonstrates the international reach, expanding methodology, and academic rigor of this academic enterprise. We trust that readers will find the series informative and engaging.

## Author contributions

CG wrote the first draft of the manuscript. RB and MT revised and finalized it. All authors contributed to the article and approved the submitted version.
